# Valid Assessment of Carbohydrate Intolerance and the Need for a Distinction to Carbohydrate Malabsorption. Comment on “Roles of Lactose and Fructose Malabsorption and Dietary Outcomes in Children Presenting with Chronic Abdominal Pain.” *Nutrients* 2019, *11*, 3063

**DOI:** 10.3390/nu12061546

**Published:** 2020-05-26

**Authors:** Karin Hammer, Johann Hammer

**Affiliations:** 1 St. Anna Kinderspital, Medical University of Vienna, 1090 Vienna, Austria; karin.hammer@stanna.at; 2 Abteilung für Gastroenterologie und Hepatologie, Universitätsklinik für Innere Medizin 3, Medical University of Vienna, 1090 Vienna, Austria

We have read with interest the recent paper by Posovszky and Roesler et al [1] that reports, based on the doctoral thesis by Dr. Roesler [2], on the possible role of carbohydrate malabsorption and dietary outcomes in children with chronic abdominal pain (CAP). A better understanding of the mechanisms underlying functional abdominal pain may improve therapeutic options beyond general dietary advices. In their conclusion, the authors confirm the questionable utility of the measurement of carbohydrate malabsorption as the basis for carbohydrate restriction diets. However, the authors fail to clearly distinguish between malabsorption and intolerance, use arbitrary and unconventional definitions of carbohydrate intolerance, and erroneously equate the pathophysiology and clinical presentation of lactose and fructose malabsorption.

Breath tests are frequently used tools for the diagnosis of malabsorption by measuring the presence of hydrogen (and methane) in the exhaled air after the ingestion of provocative doses of poorly absorbable carbohydrates such as lactose or fructose. Apart from the determination of malabsorption, a carbohydrate challenge allows for the concurrent recording of symptoms, i.e., the determination of intolerance. Carbohydrate malabsorption and intolerance are not equivalent, and intolerance does not necessarily represent the clinical consequence of malabsorption [3]. Intolerance after a carbohydrate challenge rather than malabsorption should be the main focus of clinical evaluation, as it is not malabsorption, but intolerance that correlates with clinical symptoms [4]. The treatment of carbohydrate-related symptoms by diet or enzyme replacement should not be primarily aimed at altering malabsorption, but rather at improving abdominal symptoms [5].

Having said this, it is obvious that a valid, unbiased recording of carbohydrate-induced symptoms is of eminent clinical importance [6]. Posovszky and Roesler et al have failed to standardize symptom assessment during breath tests as well as after elimination diet, allowing both patient- and doctor-related biases to influence the results. The evaluation of symptoms by inviting patients and their caregivers to report any undesignated symptom predisposes to reporting bias or cognitive bias, for example, non-standardized assessment of patients after a diet in different study centers is prone to academic bias, cognitive bias, etc. The particularly low rate of patients with lactose malabsorption (18%) in the study may furthermore be an indication of poor pretest evaluation for lactase malabsorption.

It is unlikely that none of the patients with a negative breath test (<20 ppm H_2_-increase over baseline) developed abdominal symptoms during or after the test. These patients constitute a group of patients with mainly functional abdominal pain disorders (FAPD). Visceral hypersensitivity is an important pathophysiologic feature of FAPD [7]. In adults with the irritable bowel syndrome, two of three of patients with a negative breath test had a symptom response to the ingested carbohydrate [8]. Similarly, more than half of children or adolescents with functional abdominal pain disorders had abdominal symptoms after a fructose challenge when the development of abdominal symptoms (i.e., intolerance) was evaluated by a test-specific validated questionnaire (4). Results of a study on the effect of fructose and fructose-oligomers suggest that symptoms after ingestion of carbohydrates (carbohydrate intolerance) may occur without carbohydrate malabsorption [9].

As the symptoms induced by carbohydrate ingestion are presumably linked to visceral hypersensitivity, it has been suggested to lable this entity ‘carbohydrate hypersensitivity’, thus recognizing the link to visceral hypersensitivity [10]. We are surprised that the authors consider carbohydrate intolerance as an organic instead of a functional disorder; this is unusual and cannot be substantiated by laboratory tests or imaging methods.

Fructose absorption depends on several molecular mechanisms and therefore is much more variable than lactose absorption. Malabsorption of an ingested fructose load comes about earlier when compared to an equimolar lactose load, and usually produces a relatively short H_2_-peak, whereas H_2_-exhalation persists over a longer period of time after ingestion of lactose. Furthermore, symptoms occur earlier after a fructose challenge when compared to lactose-induced symptoms (8).

In summary, we acknowledge the effort of Posovszky and Roesler et al. to study the mechanisms involved in functional abdominal pain disorder and the hassle involved in the processing, correction and editing of a thesis. Parallel to the exclusion of an organic reason for the abdominal complaints, dietary assessment remains an indispensable part of patient work-up. Testing for carbohydrate intolerance may replace testing for carbohydrate malabsorption in the future to obtain relevant clinical information. Symptom assessment during hydrogen breath measurements goes back a long time; the formerly used methods of symptom assessment do not fulfill modern, strict criteria of validated symptom assessment and should be replaced by a standardized, validated symptom evaluation to obtain a reliable and unbiased assessment of abdominal symptoms.

## References

[1] Posovszky C., Roesler V., Becker S., Iven E., Hudert C., Ebinger F., Calvano C., Warschburger P. (2019). Roles of Lactose and Fructose Malabsorption and Dietary Outcomes in Children Presenting with Chronic Abdominal Pain. Nutrients.

[2] https://oparu.uni-ulm.de/xmlui/handle/123456789/22531.

[3] Gasbarrini A., Corazza G.R., Gasbarrini G., Montalto M., Di Stefano M., Basilisco G., Parodi A., Usai-Satta P., Vernia P., Anania C. (2009). Methodology and indications of H2-breath testing in gastrointestinal diseases: The Rome Consensus Conference. Aliment. Pharmacol. Ther..

[4] Hammer V., Hammer K., Memaran N., Huber W.D., Hammer K., Hammer J. (2018). Relationship Between Abdominal Symptoms and Fructose Ingestion in Children with Chronic Abdominal Pain. Dig. Dis. Sci..

[5] Deng Y., Misselwitz B., Dai N., Fox M. (2015). Lactose intolerance in adults: Biological mechanism and dietary management. Nutrients.

[6] Hammer J., Hammer H.F. (2019). There Is an Unmet Need for Test-Specific, Validated Symptom Questionnaires for Breath Tests in Adults. Gastroenterology.

[7] Hyams J.S., Di Lorenzo C., Saps M., Shulman R.J., Staiano A., van Tilburg M. (2016). Functional Disorders: Children and Adolescents. Gastroenterology.

[8] Wilder-Smith C., Materna A., Wermelinger C., Schuler J. (2013). Fructose and lactose intolerance and malabsorption testing: The relationship with symptoms in functional gastrointestinal disorders. Aliment. Pharmacol. Ther..

[9] Major G., Pritchard S., Murray K., Alappadan J.P., Hoad C.L., Marciani L., Gowland P., Spiller R. (2017). Colon hypersensitivity to distension, rather than excessive gas production, produces carbohydrate-related symptoms in individuals with irritable bowel syndrome. Gastroenterology.

[10] Delgado-Aros S., Camilleri M. (2005). Visceral hypersensitivity. J. Clin. Gastroenterol..

